# A rare imaging case of bilateral plasmacytoma of the breast

**DOI:** 10.1259/bjrcr.20190131

**Published:** 2020-09-29

**Authors:** Joleen Kirsty Eden, Rita Borgen, Rabea Haq, Richard Dobrashian

**Affiliations:** 1 Department of Breast Imaging, East Lancashire Hospitals NHS Trust, Burnley, United Kingdom; 2 Department of Radiology, East Lancashire Hospitals NHS Trust, Burnley, United Kingdom

## Abstract

This case reports on secondary extramedullary multiple myeloma within both breasts in the absence of axillary nodal involvement and discusses the difficulty in interpretation with clinical recommendations and learning outcomes.

Differentiating plasmacytic lesions in the breast is often difficult as clinical and radiological appearances are known to mimic benignity and high-grade primary breast cancer.

Extramedullary presentation can determine progression of the disease and can necessitate cross-sectional imaging. Therefore definitive diagnosis is essential as the clinical management of the patient may be altered.

## Clinical presentation

A 73-year-old female with a history of multiple myeloma was referred for breast assessment by her haematologist for a new superficial lump with associated bruising in the upper outer quadrant of the right breast (RUOQ). Additional information detailed a history of prior pulmonary embolisms and was therefore on long-term warfarin.

**Figure 1.**
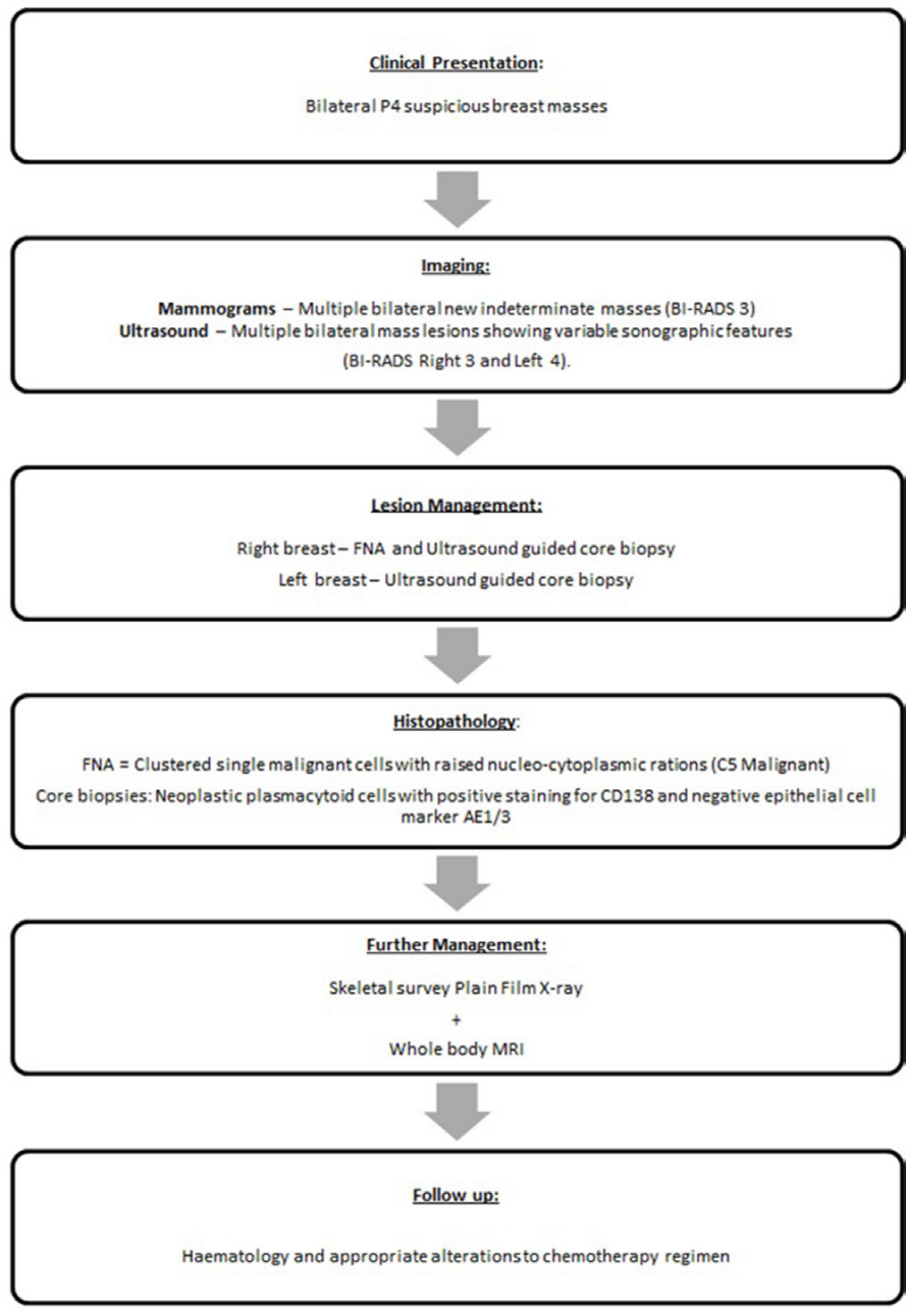
Flow chart illustrating the patient pathway and management.

Clinical breast examination revealed a suspicious lump in the RUOQ at the area of concern with overlying cutaneous bruising with two further similar masses palpated in the left outer breast (P4). The patient pathway and management is outlined in Figure 1.

## Imaging findings

### Mammograms

**Figure 2.**
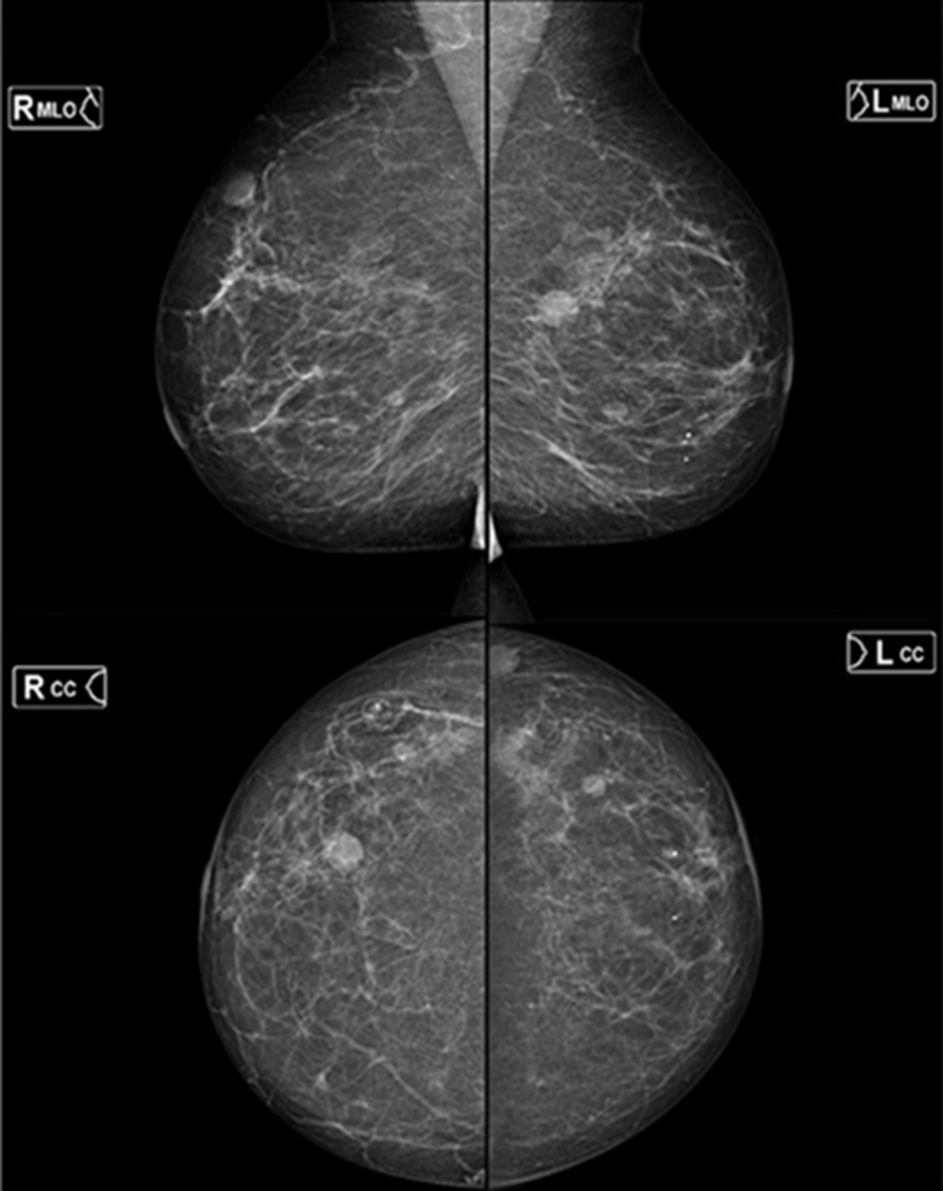
Bilateral mammograms demonstrating multiple predominantly well-defined masses with subtle areas of margin ill definition bilaterally and new compared to previous imaging of 2014. All show indeterminate appearances (BI-RADS: Right M3, Left M3).

In comparison with previous mammograms from 2014, multiple new bilateral predominantly well-defined masses with some subtle margin ill definition were identified showing indeterminate non-specific features (Figure 2) requiring further assessment. Based on the American College of Radiology, Breast Imaging Reporting and Data System (BI-RADS) classification,^1^ this was graded indeterminate M3.

### Ultrasound

**Figure 3.**
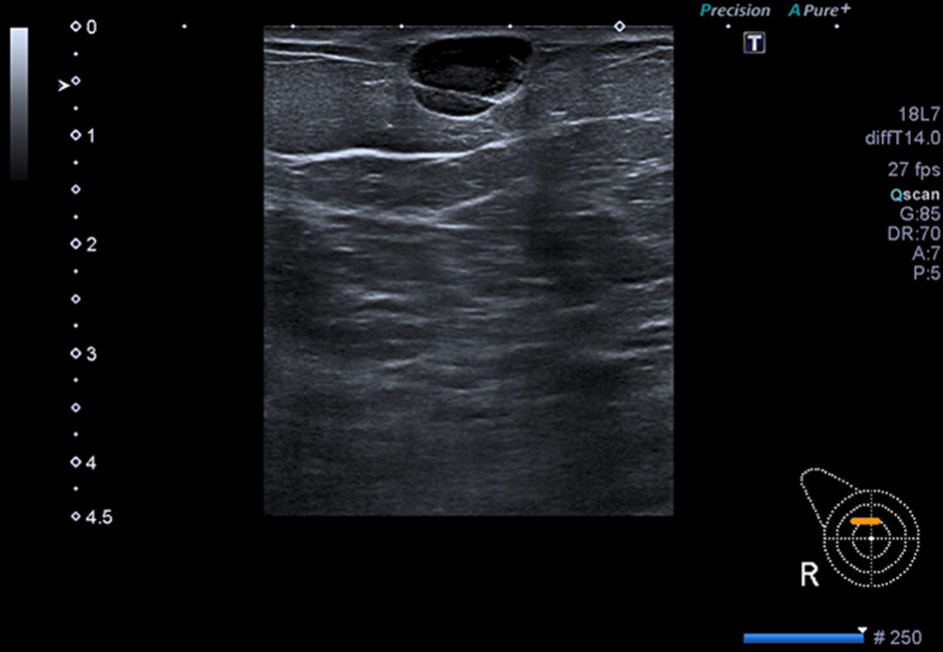
Ultrasound right breast demonstrating the index clinically palpable lesion as a well-defined “pseudo cystic mass” with low-level internal echoes, superficially located just beneath the skin corresponding to the site of overlying skin bruising (BI-RADS U3).

Ultrasound (US) of the right breast demonstrated the clinically presenting index lesion to be a superficial well-defined, “pseudocystic” lesion with low level internal echoes, BI-RADS U3 (Figure 3). Sonographically, the differential diagnoses is of an epidermal inclusion cyst, haematoma (given the overlying bruising) and fat necrosis. Percutaneous fine needle aspiration (FNA) was performed.

**Figure 4.**
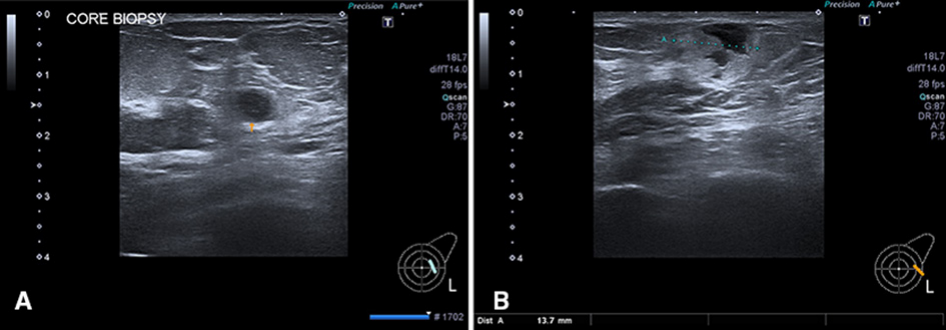
(a) US left breast demonstrates an indeterminate 7.5 mm deeper seated rounded hypoechoic solid nodule with a hyperechoic penumbra (BI-RADS U4). (b) US left breast demonstrating a superficial 14 mm partly heterogeneous lesion with peripheral solid hyperechoic component and central liquefaction mimicking fat necrosis or haematoma (BI-RADS U3).

Examination of the left breast identified two lesions, one a 7.5 mm deeper seated non-specific hypoechoic mass with a surrounding hyperechoic penumbra (BI-RADS U4, Figure 4a), and the other a 14 mm superficial part solid, part cystic lesion resembling fat necrosis or a parenchymal haematoma (BI-RADS U3, Figure 4b). Percutaneous core biopsy of the smaller more suspicious lesion was performed.

Sonographically both axillae appeared normal without any evidence of lymphadenopathy.

**Figure 5.**
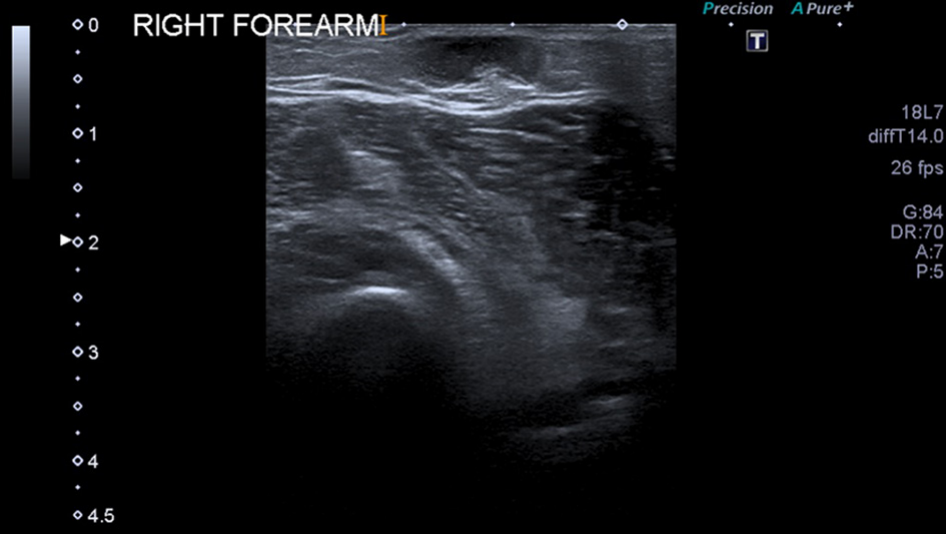
Ultrasound right Forearm demonstrating a non-specific hypoechoic superficial lesion with a hyperechoic penumbra similar to at least one of the breast lesions.

During the examination, the patient also revealed a new superficial soft tissue lump in her right forearm and ultrasound demonstrated a lesion with similar appearances to the breast lesions (Figure 5).

## Radiological differential diagnosis

Bilateral presentation of solitary or multiple well-defined round lesions can suggest benignity rather than malignancy, with a differential diagnosis of cysts or fibroadenomas. In this case, the differing sonographic features of each lesion demonstrated the spectrum of imaging appearances with differential diagnoses including epidermal inclusion cysts, fat necrosis, haematoma, metastases and primary breast malignancy. The soft tissue lesion found within the right forearm had similar appearances to the breast lesions and this increased suspicion of metastases.

The characteristic imaging appearances associated with epithelial breast carcinoma include irregular masses, architectural distortion and microcalcifications. None of the aforementioned were identified in this case. Secondary changes often seen in primary breast carcinomas such as skin tethering, nipple retraction and peau d’orange were all absent.

No history of trauma to the breast was ascertained although given the history of warfarin use, spontaneous breast haematomas was certainly a consideration.

Given the clinical history of myeloma; multiple benign appearing masses on imaging indicated a strong suspicion of metastatic extramedullary progression.

## Histopathology

**Figure 6.**
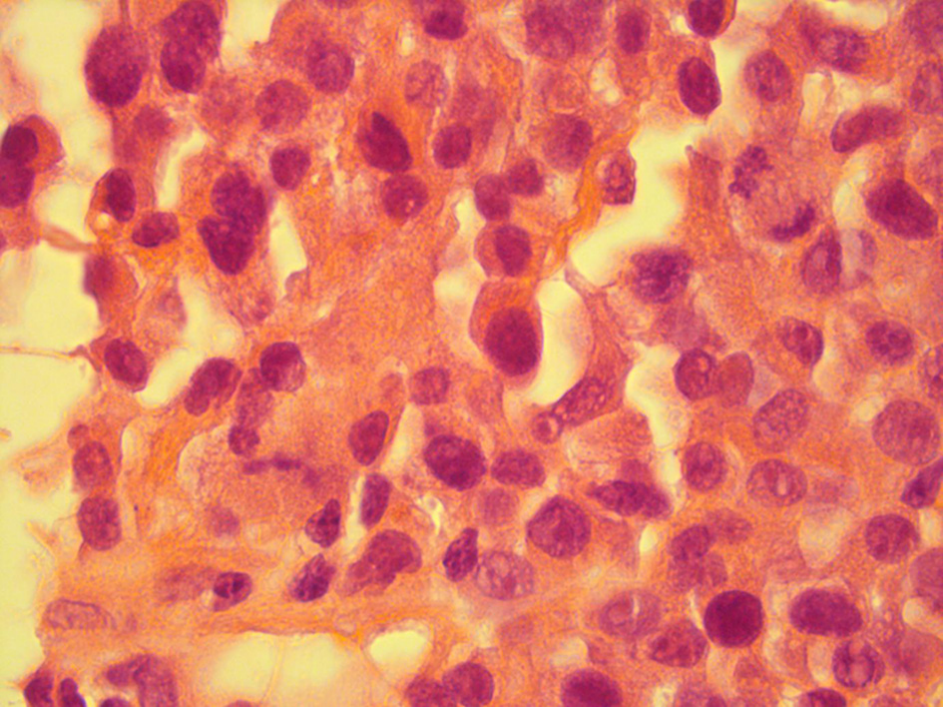
Histopathology breast core H&E stain 40x demonstrating pleomorphic plasmacytoid cells with abnormal mitotic activity.

**Figure 7.**
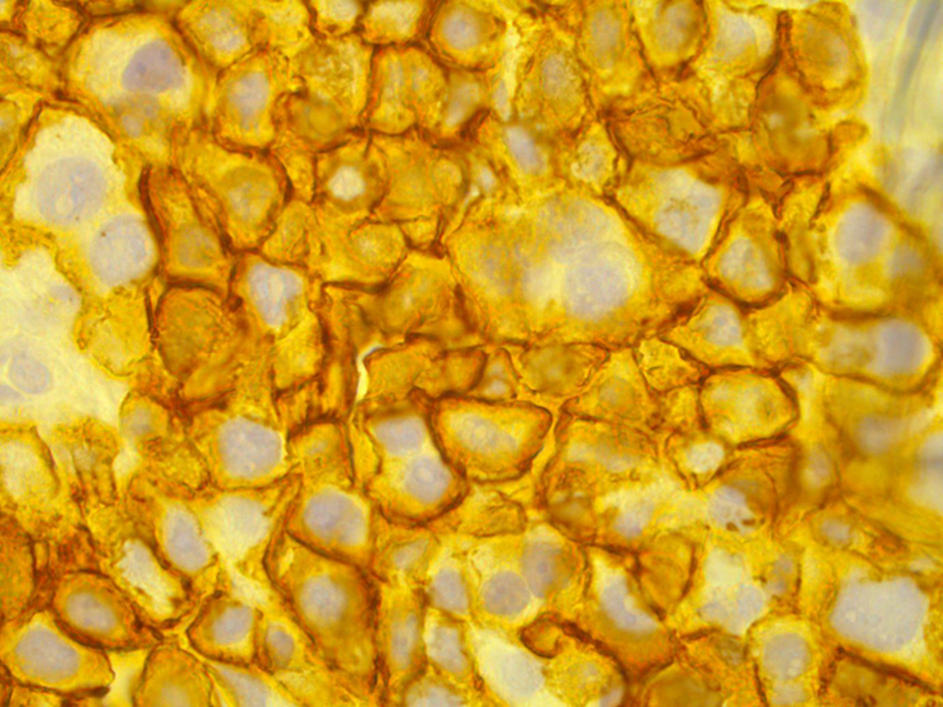
Histopathology slide demonstrating positive staining of plasmacytoid cells with plasma cell marker CD138 confirming diagnosis of plasma cell myeloma.

FNA of the right breast superficial index lesion confirmed the presence of clustered and single malignant cells with raised nucleo-cytoplasmic ratios, prominent nucleoli and clumped chromatin (C5-malignant). Subsequent core biopsy demonstrated heavy infiltration by neoplastic plasmacytoid cells forming sheets of moderately pleomorphic cells with enlarged rounded eccentrically placed nuclei highly suspicious of myeloma (B5–malignant, Figure 6). Positive staining of plasmacytoid cells with plasma cell marker CD138 confirmed the diagnosis of plasma cell myeloma (Figure 7). Additionally, there was negative staining of plasmacytoid cells with epithelial cell marker AE1/3.

Core biopsy of the suspicious left breast lesion showed identical features.

## Further management

**Figure 8.**
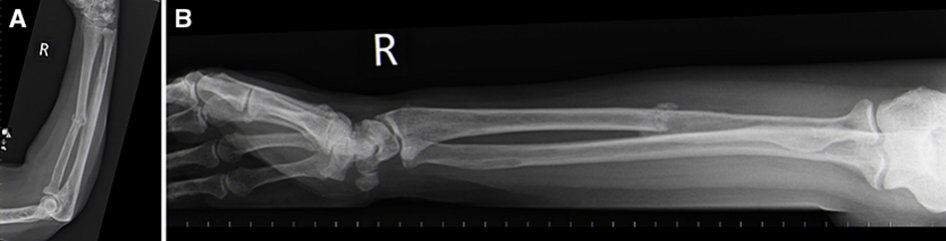
(a) and (b) plain film digital X-ray—Radius/Ulna demonstrating the spectrum of myeloma changes on plain film. Multiple well-defined punched out medullary lytic lesions with endosteal scalloping and a more permeative lytic lesion mid radial shaft with a pathological fracture.

**Figure 9.**
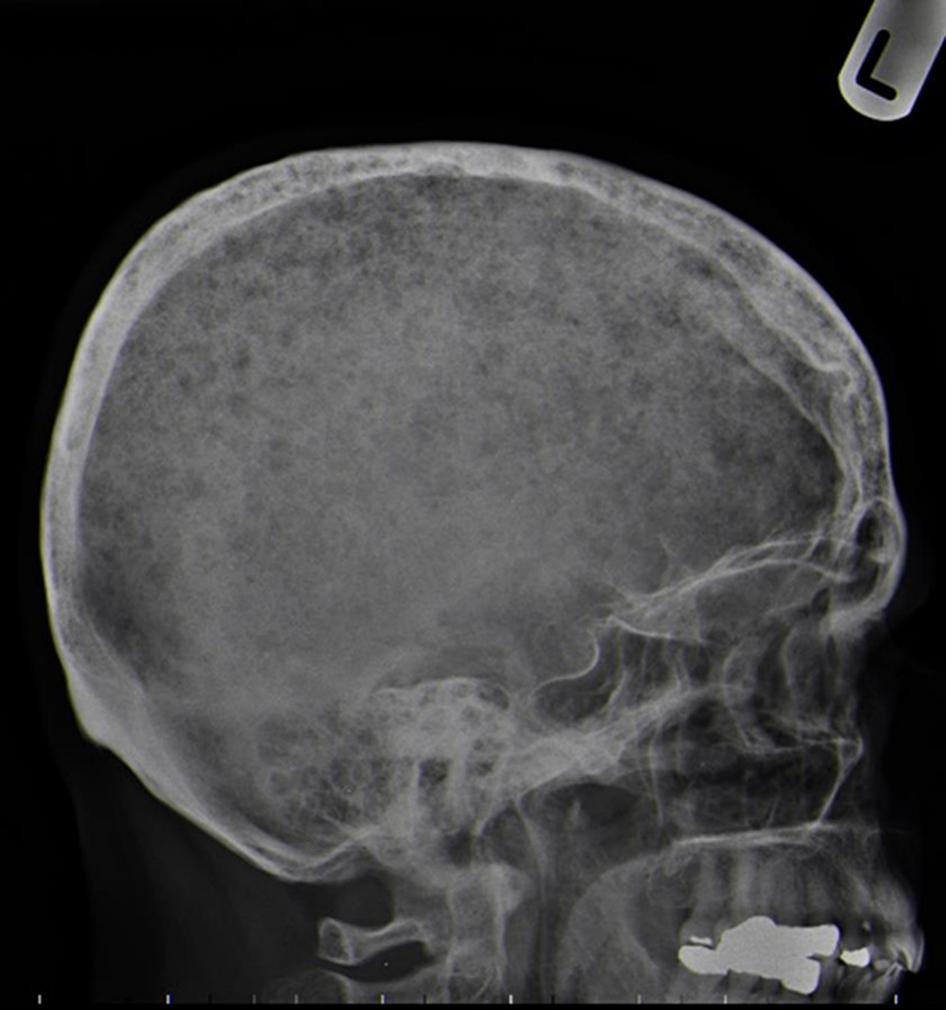
Plain skull film digital X-ray—demonstrating classic multiple lytic calvarial lesions often termed “rain drop” lesions or “pepper pot” skull.

The patient underwent a skeletal survey to assess the potential extent of myeloma. Lytic lesions were found in the radius and ulna with an associated pathological fracture (Figure 8a and b). Multiple lesions were also noted in the skull vault demonstrating the “raindrop” or “pepper pot” sign, radiologically classic of myeloma (Figure 9).

**Figure 10.**
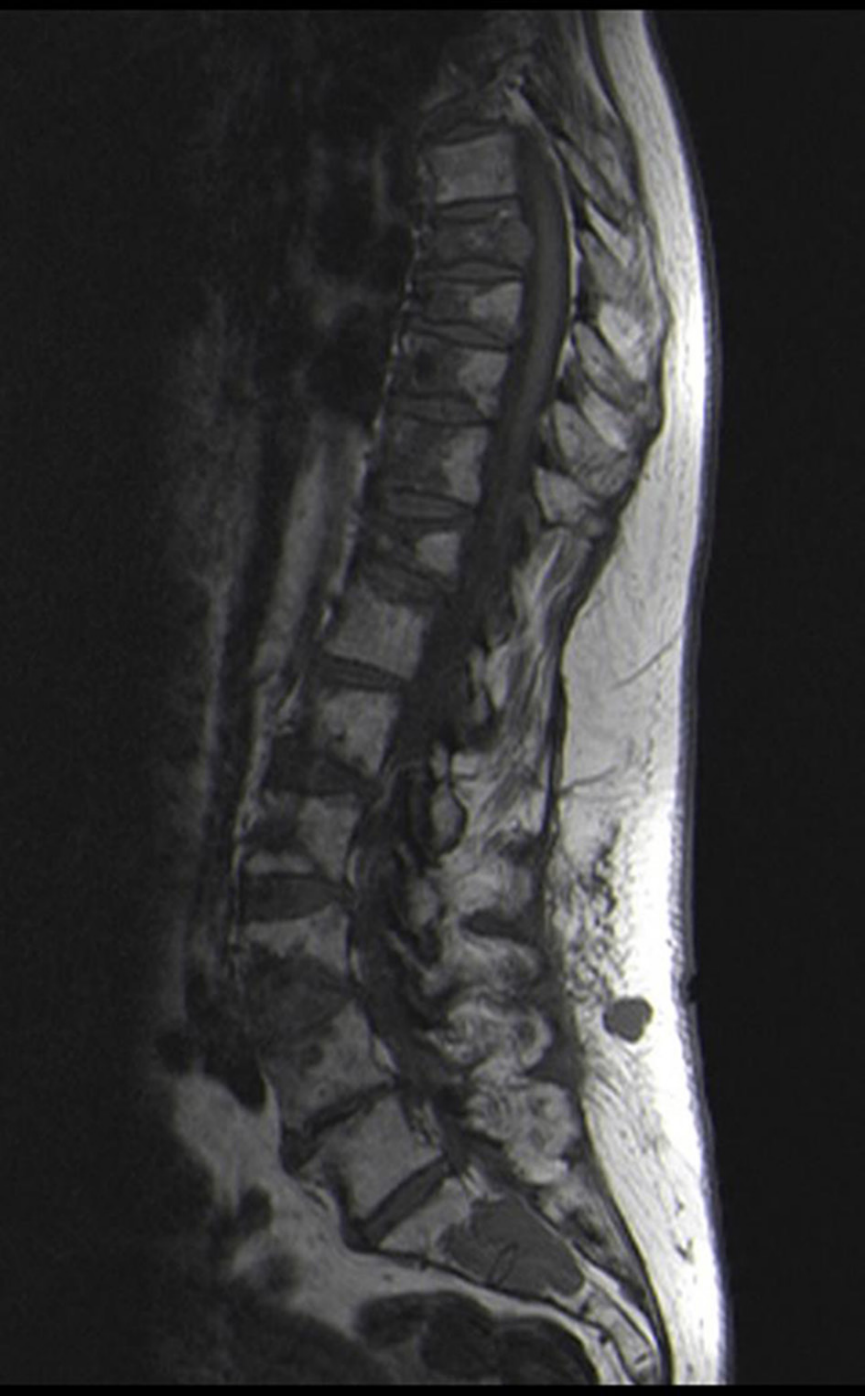
Sagittal T1W MRI of the thoraco-lumbar spine demonstrating widespread vertebral marrow deposits and associated multiple wedge fractures of varying severity throughout the thoracic and lumbar spine. Multiple soft tissue deposits in the subcutaneous fat overlying the lumbar spine representing extra skeletal disease and there is a further area adjacent to the right iliac blade deep to the gluteal muscle.

MRI is now the gold standard for imaging the skeleton to assess extramedullary progression and in this case showed the marrow and soft tissue deposits very eloquently (Figure 10).

## Follow-up

Following additional imaging demonstrating disease progression, haematological review was undertaken and appropriate alterations to chemotherapeutic management made.

## Discussion

Multiple myeloma is a monoclonal proliferative disease of the plasma cells within bone marrow. Worldwide in 2016 there were 138,509 cases reported.^2^ In the UK, 2% of cancers reported are myeloma which equates to approximately 15 new cases every day. It is the 19th most common cancer. Since the early 1990s in the UK incidence has increased by almost a third.^3^ Successful treatment is dependent on the extension of the disease at diagnosis, and although considered incurable, treatment options have the potential to prolong survival expectancy. Therefore, clinical responsiveness and early diagnosis are crucial for patient outcomes.

The spectrum of disease includes solitary bone plasmacytoma, extramedullary plasmacytoma without bone marrow infiltration, multiple myeloma with extramedullary manifestations and plasma cell leukaemia.

Plasmacytoma is a localized form of myeloma characterized by a collection of malignant monoclonal plasma cells which is often associated with lytic lesions of the bone, however may be extramedullary and early literature suggests that 3% of cases may involve soft tissue.^4^ Extramedullary disease is defined as the presence of malignant plasma cells external to bone marrow in patients with a diagnosis of myeloma. It is associated with poor prognosis and is thought to be secondary to haematological spread.^5^ Highly vascularized soft tissue plasmacytic lesions may arise in the skin, liver, breast or kidneys, however can arise in any organ.^6^

Plasmacytoma of the breast is very rare with very few cases reported within the literature. Between 1928 and 2009, only 63 cases of breast plasmacytoma have been reported equating to approximately 15 cases per year, most of which are unilateral.^7,8^ Mammography and sonographic features vary considerably, with 2% of cases completely occult mammographically.^8^ Typically, lesions are often well-defined sometimes with irregular or ill-defined margins without spiculations or microcalcification.^9^

Sonographically, lesions appear solid or “pseudocystic” with hypoechoic, hyperechoic or mixed echogenicity and often have well-defined or irregular margins. Lesions in this case were superficially located in the subcutaneous tissue and within the deep stroma. Variation in distal acoustic effect can occur with enhancement or shadowing and can therefore be unhelpful in analysis of the lesion. Doppler flow can assist in indicating increased vascularity within the lesion and shear wave elastography can show stiffness. These are all non-specific features.

The imaging features in this case are very non-specific with mammography showing multiple predominantly well-defined masses and ultrasound demonstrating a variety of appearances from well-defined “pseudocystic,” through to heterogeneously solid with an inflammatory penumbra and part solid, part cystic resembling a broad spectrum of pathology including epidermoid inclusion cysts, fat necrosis, haematoma, fibroadenomas, metastases and primary high-grade breast cancer. The absence of associated microcalcification however is a valuable feature to aid in differentiation from primary breast cancer. The well-defined nature likely stems from the haematogenous route taken to the breast in most cases with a stromal location. The “pseudocystic” and complex cystic appearance is harder to explain other than tumour biogenesis and rapidity of growth.

Clinically breast plasmacytoma often presents as a palpable mass, occasionally with inflammatory changes such as skin thickening which may suggest abscess or inflammatory carcinoma.^10^ Skin thickening was not seen in this case however bruising to the overlying skin was present likely due either to warfarin use or associated thrombocytopenia from marrow infiltration leading to blood coagulation abnormalities.^11^ Never the less this could mislead into thinking the breast changes were due to haematoma formation or fat necrosis.

Extramedullary disease usually manifests itself in the lungs, gastrointestinal tract or bladder, it is very rare to present within the breast.^12^ Breast plasmacytoma can be unilateral or bilateral and although rare usually occur in the setting of advanced multiple myeloma.

There is little peer-reviewed literature discussing breast plasmacytoma due to the rare presentation. Surov et al (2010) reviewed five cases within their institution between 1997 and 2008 with an additional 48 patients found in the literature between 1988 and 2010.^13^ Of the 53 cases, eight patients (15%) had a primary breast plasmacytoma, the other 45 cases (85%) had an extramedullary presentation of more widespread multiple myeloma. Most of the cases had a solitary presentation (66%) rather than multiple (34%), however the size of lesion ranged from 8 to 90 mm.

As with all focal breast lesions triple assessment is best practice. Cytology with FNA has been shown to demonstrate plasma cells at different stages of maturation; therefore core biopsy is not always necessary however adequate sampling is required to differentiate from primary carcinoma.^14^

Lymphadenopathy associated with breast plasmacytoma is rare; however a case of a 78-yearold male has been reported with lymph node involvement and extracapsular extension of the tumour.^15^

The value of MRI of the breast for plasmacytoma is subjective as imaging features are often inconclusive. A case reported by Kocaoglu et al in 2003 concluded the benign features associated with plasmacytoma may be misleading despite MR contrast enhancement.^16^ This correlated with the literature suggesting that MRI is unnecessary providing mammography and ultrasound is combined with histopathology.

If extramedullary involvement is suspected recommendations from the International Myeloma Working group suggest MRI or CT combined with FDG-PET is indicated for the assessment of disease extent and monitoring of treatments.^17^ MRI/CT may identify osteolytic lesions which even when unifocal should be regarded as meeting the CRAB (hypercalcaemia, renal failure, anaemia and bone lesions) criteria, regardless of whether they are seen on plain-film skeletal radiographs.^17^ Thus confirming a diagnosis of multiple myeloma and altering patient management.

Medullary myeloma usually is identified radiologically by lytic lesions due to the dominance of osteoclast activity with suppression of osteoblast formation.^18^ Destruction of the cortex can lead to local invasion of the soft tissues however metastatic soft tissue deposits are often an advanced presentation of myeloma and define extramedullary disease which correlates with the findings in our case.

## Learning points

1. Imaging appearances are varied ranging from superficial cystic type lesions to solid nodules with benign or ill-defined margins. Lymph node involvement is rare but can be present and MRI for breast assessment can be misleading. Presentation within the breast is unusual but can appear bilaterally and combined with benign features should not be falsely reassuring. Breast imagers need to be aware of this when imaging patients with a clinical history of myeloma.
2. Demonstrating extramedullary soft tissue involvement can indicate disease progression and necessitate further cross-sectional imaging, potentially changing clinical chemotherapeutic management.
3. Haematology clinicians need to be aware of the presentation which may manifest in the breast and include routine breast examination at follow-up clinical appointments.
4. Triple assessment approach is advocated even when imaging appearances look benign. Core biopsy is preferred although adequate fine needle aspiration can be sufficient.
